# In Vivo Plasticity at Hippocampal Schaffer Collateral-CA1 Synapses: Replicability of the LTP Response and Pharmacology in the Long-Evans Rat

**DOI:** 10.1155/2020/6249375

**Published:** 2020-11-12

**Authors:** A. Ahnaou, E. White, R. Biermans, N. V. Manyakov, W. H. I. M. Drinkenburg

**Affiliations:** Department of Neuroscience, Janssen Research & Development, Janssen Pharmaceutica NV., Turnhoutseweg 30, B-2340 Beerse, Belgium

## Abstract

Broad issues associated with non-replicability have been described in experimental pharmacological and behavioral cognitive studies. Efforts to prevent biases that contribute to non-replicable scientific protocols and to improve experimental rigor for reproducibility are increasingly seen as a basic requirement for the integrity of scientific research. Synaptic plasticity, encompassing long-term potentiation (LTP), is believed to underlie mechanisms of learning and memory. The present study was undertaken in Long-Evans (LE) rats, a strain of rat commonly used in cognitive behavioral tests, to (1) compare three LTP tetanisation protocols, namely, the high-frequency stimulation (HFS), the theta-burst stimulation (TBS), and the paired-pulse facilitation (PPF) at the Schaffer collateral-CA1 stratum radiatum synapse and to (2) assess sensitivity to acute pharmacology. *Results*: (1) When compared to Sprague-Dawley (SD) rats, the HFS using a stimulus intensity of 50% of the maximum slope obtained from input/output (I/O) curves elicited lower and higher thresholds of synaptic plasticity responses in SD and LE rats, respectively. The 2-train TBS protocol significantly enhanced the LTP response in LE rats over the 5- and 10-train TBS protocols in both strains, and the 5 × TBS protocol inducing a subthreshold LTP response was used in subsequent pharmacological studies in LE rats. The PPF protocol, investigating the locus of the LTP response, showed no difference for the selected interstimulus intervals. (2) Scopolamine, a nonspecific muscarinic antagonist, had a subtle effect, whereas donepezil, an acetylcholinesterase inhibitor, significantly enhanced the LTP response, demonstrating the sensitivity of the TBS protocol to enhanced cholinergic tone. MK-801, a noncompetitive N-methyl-D-aspartate (NMDA) antagonist, significantly reduced LTP response, while memantine, another NMDA antagonist, had no effect on LTP response, likely associated with a weaker TBS protocol. PQ10, a phosphodiesterase-10 inhibitor, significantly enhanced the TBS-induced LTP response, but did not change the PPF response. Overall, the results confirm the strain-dependent differences in the form of synaptic plasticity, replicate earlier plasticity results, and report effective protocols that generate a relatively subthreshold margin of LTP induction and maintenance, which are suitable for pharmacology in the LE rat strain mainly used in cognitive studies.

## 1. Introduction

Cognitive function can be defined as cerebral activities and processes involved in acquiring new knowledge and understanding thoughts, experiences, and senses, encompassing attention, memory, perception, language comprehension, decision making and judgment, and executive control functions [1]. Locally, these complex processes rely on the exchange of information between critical brain regions through synaptic transmission. Synaptic plasticity, an activity-dependent modification of the strength and/or efficacy of synaptic transmission at synapses is thought to underlie the ability of our brain to encode, process, and store new, incoming information [2]. Synaptic plasticity encompasses two main processes: long-term potentiation (LTP) and long-term depression (LTD) characterized as a persistent increase and decrease in synaptic strength, respectively. Both processes interact and alter synaptic strength and connectivity in response to specific activity patterns [3]. Synaptic plasticity induces changes both pre- and postsynaptically. Presynaptically, changes in neurotransmitter release, exocytosis mediators, and the action of neuromodulatory transmitters can all alter synaptic strength and transmission [4]. Postsynaptically, alterations in glutamatergic receptors, signal transduction pathway activation, gene activation, synthesis of new proteins, spine growth, and synaptic pruning contribute to synaptic plasticity mechanisms [4].

LTP is the most popular and widely studied model of synaptic plasticity, as many of its properties reflect the cellular processes underlying learning and memory functions [5–7]. LTP, a persistent increase in synaptic strength, is most commonly induced by combined activation of *α*-amino-3-hydroxy-5-methyl-4-isoxazolepropionic acid (AMPA) and N-methyl-D-aspartate (NMDA) glutamate receptors. LTP can be split into short-term and long-term plasticity; the short-term involves Ca^2+^-dependent activation of protein kinases, phosphorylation of AMPA receptors, and translocation of internalized AMPA receptors to the postsynaptic membrane [8]. Long-term changes encompass increases in gene transcription and protein synthesis through inhibition of phosphodiesterase (PDE), which increases the concentration of cAMP and/or cGMP leading to enhanced LTP expression [9]. Increased PDE activity has been implicated in both aging and neurodegenerative diseases such as Alzheimer's disease (AD), making them increasingly important pharmacological targets. PDE4 inhibition has been found to reverse MK-801-induced LTP deficit [10]. PDE2A inhibition has been shown to enhance the presynaptic form of LTP [11]. PQ10, a phosphodiesterase-10 inhibitor, targets both cAMP and cGMP pathways.

Cholinergic inputs from the medial septal area onto the hippocampus are thought to play important modulatory roles in synaptic plasticity and cognition [12]. The hippocampus contains two main types of acetylcholine receptors (AChRs): nicotinic receptors (nAChRs), which are ligand-gated ion channels, and muscarinic receptors (mAChRs), which are G-protein-coupled receptors [13]. Cholinergic input is thought to facilitate LTP through mechanisms such as the enhancement of NMDA receptor function via inhibition of small conductance-activated potassium channels, modulation of GABAergic inhibition of pyramidal neurons, enhanced postsynaptic excitability, action potential generation, and postsynaptic blocking of K^+^ conductance [12]. The regulation of the cholinergic system is one of the treatments of neurodegenerative diseases such as AD. By stimulating the cholinergic system by inhibiting acetylcholinesterase and enhancing the activity of the NMDA system and reversing the activity, the mechanism tends to increase the activity of the pyramidal neurons in the CA1 hippocampal region [14, 15].

Laboratory mice and rats are the main mammalian models used in experimental pharmacological and behavioral cognitive research; however, preclinical studies are prone to broad issues related to non-replicability and non-reproducibility [16, 17]. For instance, strain-related differences in animals performing cognitive tasks have been shown to affect the outcome of experiments and are responsible for discrepancies obtained following pharmacological, behavioral, and environmental experimental manipulations. Therefore, behavioral phenotyping of baseline performance of laboratory animal strains has a great implication for the selection of an animal strain and protocol best suited for pharmacology testing in a given paradigm. In neurobiological and behavior studies in the rat, strain-dependent differences in sensitivity to pharmacologic agents showed that outbred pigmented Long-Evans (LE) rats may be more appropriate than other strains in tests of motor and cognitive functions [18, 19]. LE rats were reported to be more successful in the autoshaping of a lever press and a two-object discrimination test but worse in a two-island water maze task compared to outbred albino strains and Wistar and Sprague-Dawley (SD) rats [20, 21].

There are, however, relatively few studies comparing the potential strain-related differences in activity-dependent neuroplasticity in rats [22]. Only one previous study has directly compared LTP electrophysiology in the medial perforant pathway of the dentate gyrus between LE and SD rats [23]. LE rats exhibited greater LTP induction and persistence, a finding that may relate to the elevated excitability of the granule cells, although variations in other factors including the level of neuromodulatory transmitters, such as noradrenaline, could also play a role in both the excitability change and the LTP enhancement [24–26]. Based on this evidence, LTP response and expression may vary with rat strains and protocols, making it important to replicate and validate LTP induction protocols in a given animal strain.

This study is aimed at (1) comparing the HFS and TBS LTP induction protocols between LE and SD rats and (2) evaluating the effects of pharmacological modulation of critical signaling on hippocampal posttetanic potentiation (PTP), short-term potentiation (STP), and long-term potentiation (LTP) at the SC-CA1 hippocampal circuit in anesthetized LE rats using donepezil (acetylcholinesterase inhibitor), scopolamine (antagonist of muscarinic receptors), MK-801, memantine (noncompetitive NMDA antagonists), and PQ10 (PDE 10 inhibitor).

## 2. Materials and Methods

### 2.1. Animal Husbandry

All experimental procedures were conducted in strict accordance with the guidelines of the Association for Assessment and Accreditation of Laboratory Animal Care International (AALAC) and with the European Communities Council Directive of 24th November 1986 (86/609/EEC) and were approved by the local ethical committee. Male LE rats and SD rats (170-280 g) were acquired from Charles River Laboratories, Germany. Female animals were not used due to cyclical interference enhancing the levels of oestrogen, which was found to facilitate LTP. The rats were group housed in individually ventilated cages (IVC) with food and drink *ad libitum*. The IVC racks were maintained in a controlled environment that was sound attenuated, had a 22 ± 2°C ambient temperature, had a relative humidity of 60%, and had a 12 : 12 light-dark cycle with a light intensity of 100 lux.

### 2.2. Surgery and Electrode Placement and Recordings

The rats were anesthetized with urethane (1.5 g/kg, intraperitoneally (ip)), which could be supplemented by injections of 0.1-0.2 mg/kg (mg/kg) if a rat was still displaying a withdrawal reflex following foot pinches. The rats were fixed in a motorized stereotaxic frame (Stoelting, Wood Dale, USA). A recording electrode (tungsten wire, 75 *μ*m diameter, impedance 10 k*Ω*) was inserted into the stratum radiatum of the CA1 (CA1: AP -4.2 mm; ML -4.0 mm) and was made to descend into the tissue at 0.2 mm/min until a theta rhythm could be seen. The bipolar stimulating electrode (tungsten twisted wires, WPI, 75 *μ*m diameter, impedance 10 k*Ω*) was inserted into the Schaffer collateral pathway (SC: AP -3.4 mm; ML 2.5 mm).

### 2.3. Input/Output Functions

Before the full LTP experiment, the correct placement of SC-CA1 implants was finely adjusted by altering the depth of both stimulation and recording electrodes in 10 *μ*m increments for optimal evoked field postsynaptic potentials (fEPSP) through the oscilloscope using single square pulses (200 *μ*s, 100 *μ*A). For stimulations, the settings of the stimuli were determined using a LabVIEW homemade software, and stimuli were sent via a data acquisition board linked to a constant current isolator unit (Multi Channel System MC STG4002). The evoked fEPSP responses of the SC were passed through an BioSemi ActiveTwo electrode amplifier (differential amplifier, Netherlands) and digitized at 3 kHz. This response was digitized at a sampling rate of 3 kHz, and data were saved in a file for offline analysis.

At the beginning of each experiment, an input/output (I/O) curve was generated (average of 3 pulses of 200 *μ*s from 1 to 8 V in steps of 1 V at 0.033 Hz) to determine the voltages used during the LTP experiment.

### 2.4. Defined Inclusion/Exclusion Criteria at SC-CA1 Synapses

For all experiments, recordings were performed for at least half an hour to obtain a stable EEG prior to the start of the experiments. The fEPSPs are single negative deflections with the onset and peak latency of 8-12 ms after a stimulation artefact; they have a maximum amplitude of 2 mV and a maximum slope of 450 *μ*V/ms. Preparations with a maximum fEPSP amplitude of 2 mV and a slope plateau of >500 *μ*V/ms at the maximum stimulus current were rejected. Field potentials generated to produce the input/output curves indicate similar thresholds for inducing baseline synaptic efficacy in the study groups. The normalized baseline fEPSP slope prior to pharmacological treatment does not exceed ±10%. The magnitude of potentiation was expressed as the percentage of increase in the fEPSP slopes at the time points after tetanic stimulation relative to the slopes averaged over the 30 min baseline period prior to tetanus stimuli. Induction of LTP was defined as an increase in fEPSP slope that is maintained above 120% relative to baseline for 2 hours following tetanisation protocols. Artefacts should be <50% of the data recorded; in the case of a higher artefact occurrence, the animal would be completely “discarded due to technical issues.” Consequently, missing data points should in such cases not be considered in the average response.

### 2.5. In Vivo Electrophysiology LTP Protocols

#### 2.5.1. High-Frequency Stimulation (HFS)

**Figure 1:**
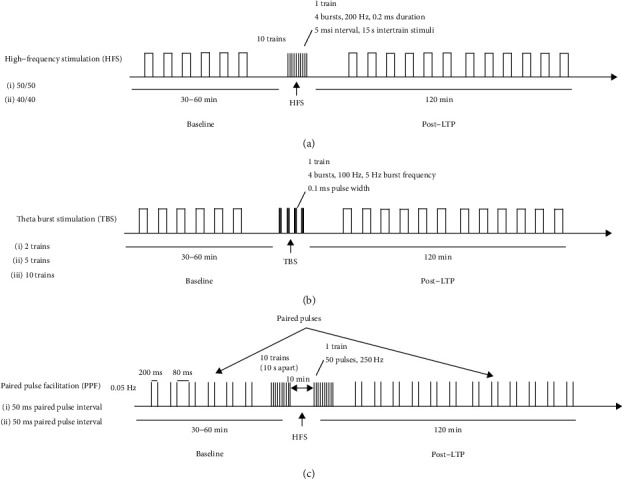
Schematic presentation of electrical stimulation protocols that induce LTP at the SC-CA1 synapses in anesthetized rats. A high-frequency stimulation protocol consists of 10 trains, 1 train consisting of 20 pulses at 200 Hz with (a) a 2 s interval between each train, (b) TBS (2 or 5 trains with 5 bursts per train of 4 pulses at 100 Hz, 200 *μ*s), and (c) paired pulses (200 ms, 0.05 Hz) delivered at different lengths of interstimulus intervals of 50 ms and 30 ms before and after the standard 200 Hz HFS protocol.

Baseline synaptic transmission was measured by delivering pulses at 0.033 Hz for 60 minutes before tetanisation. Posttetanisation fEPSPs were recorded in LE and SD rats for the following 2 hours using a previously described protocol [27], in which LTP response was induced with 10 trains, 1 train consisting of 20 pulses at 200 Hz with a 2 s interval between each train (Figure 1(a)). Afterwards, two HFS protocols were compared in LE rats: the 50/50 HFS (baseline, tetanisation, and posttetanisation pulses at 50% of the I/O curve max) and the 40/40 HFS (baseline, tetanisation, and posttetanisation pulses at 40% of the I/O curve max).

#### 2.5.2. Theta-Burst Stimulation (TBS)

Three different TBS protocols were used to compare LTP responses in LE and SD rats. 2 × TBS is composed of 2 trains with 5 bursts per train of 4 pulses at 100 Hz for 200 *μ*s, with 50% of the I/O curve maximum and an interburst interval of 200 ms (5 Hz). 5 × TBS is composed of 5 trains with 5 bursts per train of 4 pulses at 100 Hz for 200 *μ*s, with 50% of the I/O curve maximum and an interburst interval of 200 ms (5 Hz). 10 × TBS is composed of 10 trains with 5 bursts per train of 4 pulses at 100 Hz for 200 *μ*s, with 50% of the I/O curve maximum and an interburst interval of 200 ms (5 Hz) (Figure 1(b)).

#### 2.5.3. Paired-Pulse Facilitation (PPF)

PPF was used to determine the loci of LTP in the LE rats. The protocol consisted of paired pulses (200 ms, 0.05 Hz) delivered before and after the standard 200 Hz HFS protocol. Two different lengths of interstimulus intervals (ISIs) of 50 ms and 30 ms were compared (Figure 1(c)).

### 2.6. Drugs

Drugs were administered subcutaneously (sc) or intraperitoneally (ip) 30 minutes prior to tetanisation at a volume of 1 ml/100 g body weight and compared to vehicle. Three critical pathways for LTP function were pharmacologically altered to ensure that the protocols were sensitive to both increases and decreases in LTP response. The glutamatergic pathway using MK-801 (5 mg/kg, ip), a noncompetitive NMDA antagonist used to inhibit glutamatergic signaling, was administered 30 minutes before tetanisation. The cholinergic pathway uses scopolamine (0.64 mg/kg, sc), an antimuscarinic to inhibit cholinergic signaling, and donepezil (1 mg/kg, sc), an acetylcholinesterase inhibitor used to enhance cholinergic signaling. The intracellular signaling pathway using PQ10 (3 mg/kg, sc), a phosphodiesterase enzyme 10 inhibitor used to enhance cAMP and cGMP signaling pathways, was tested with both TBS and PPF protocols.

### 2.7. Histology

At the end of the electrophysiological study, three 30 sec electrical pulses of 500 *μ*A were delivered to produce a lesion at the end tip of the stimulation and recording electrodes and brains were harvested for histological verification of electrode placement. Brain sections (20 *μ*m) were examined using a light microscope. Animals with incorrect electrode placement were excluded from the study.

### 2.8. Data Analysis

The slope of the fEPSP was calculated using a linear fit least square analysis on the 80% interval between the artefact end and the negative peak. fEPSP slopes were obtained every 2.5 min as an average of 5 responses at 0.033 Hz and were then expressed as mean percentage change from baseline (defined as the last 30 min prior to tetanisation). The group-averaged time course of fEPSP together with 95% confidence intervals around the mean are shown.

Short-term potentiation (STP) value was defined as an averaged value in intervals of 1-10 minutes after HFS or TBS. Long-term potentiation (LTP) value was defined as an averaged value in intervals of 100-120 min after HFS or TBS. And posttetanic potentiation (PTP) was defined as an immediate value after HFS or TBS. Results are presented as bar plots for PTP, STP, and LTP with whiskers showing 95% confidence intervals around the means. Statistical comparison of PTP, STP, and LTP was performed using analysis of variance (ANOVA) followed by a post hoc test (Dunnett's test). *p* < 0.05 was considered statistically significant. In case of significance, differences are indicated by asterisks on box plots(^∗^*p*value < 0.05; ^∗∗^*p*value < 0.01; and ^∗∗∗^*p*value < 0.001).

In order to assess such longitudinal changes such as speed of degradation after application of HFS or TBS, mixed-effect modelling was applied. If needed, time after HFS or TBS was log transformed as *t*_new_ = ln(1 + *k* · *t*_old_), where *t*_old_ is a real time expressed in minutes. This transformation allowed linearizing the data. Coefficient *k* was adjusted for each data individually. Then, fEPSP relative to baseline (%) variable was modelled as *t*_new_∗group + (1 | animal), where group variable is a categorical variable describing different conditions assessed during the test. The effect of group variable on a slope of the model (to assess differences in degradation of fEPSP responses in time) was tested. *p* < 0.05 was considered statistically significant.

## 3. Results

### 3.1. LTP Response Was Significantly Higher in LE than SD Rats and Did Not Differ Much when Using 50/50 and 40/40 HFS Protocols in LE Rats

**Figure 2:**
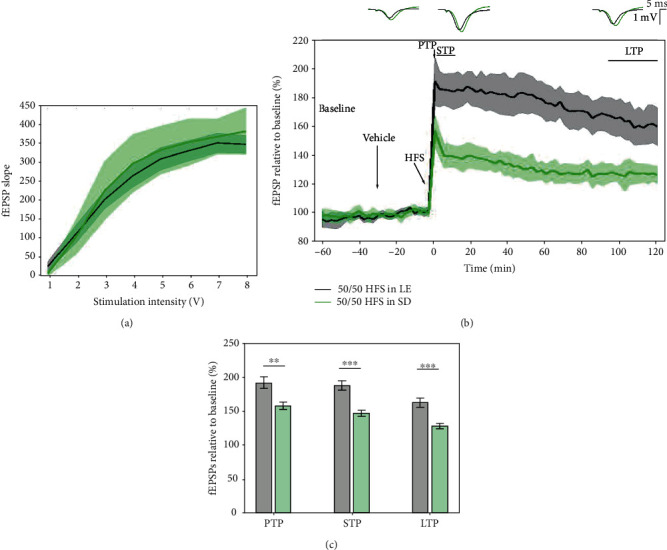
Differential synaptic LTP response to high-frequency stimulation in LE and SD rats. (a) Average input/output curves between stimulation voltage and fEPSP slope show no significant difference in basal synaptic excitability. (b) LTP responses to the 50/50 HFS protocol in LE rats (black, *n* = 11) were significantly higher than in SD rats (green, *n* = 8) through the whole 2-hour posttetanisation. Data are presented as means ± 95%confidenceintervals. Outsets above the LTP curve represent average waveform field potentials during 30 min baseline prior to tetanisation and 0-30 min and 90-120 min posttetanisation. Horizontal bar: 1 mV; vertical bar: 5 ms. (c) PTP, STP, and LTP responses are presented as means ± 95%confidenceintervals; ^∗∗^*p*value < 0.01 and ^∗∗∗^*p*value < 0.001).

As LE rats are the most commonly used strain in cognitive-based tasks, it is expected that they may display increased synaptic plasticity in comparison to other strains, such as SD rats. Therefore, different LTP induction protocols may be more suitable for pharmacological testing in these two different strains. The average input/output curves between stimulation voltage and fEPSP slope show no significant difference in basal synaptic excitability (Figure 2(a)). Statistical analysis revealed a significant effect of HFS tetanisation on PTP (LE: 191.45 ± 16.883%; SD: 157.114 ± 10.341%; *p* = 0.007), STP (LE: 187.08 ± 12.348%; SD: 146.217 ± 8.366%; *p* < 0.001), and LTP (LE: 162.051 ± 12.521%; SD: 127.307 ± 6.842%; *p* < 0.001) (Figure 2(c)). When assessing degradation of fEPSP, a significant difference in the model's slopes was found (*p* < 0.01).

**Figure 3:**
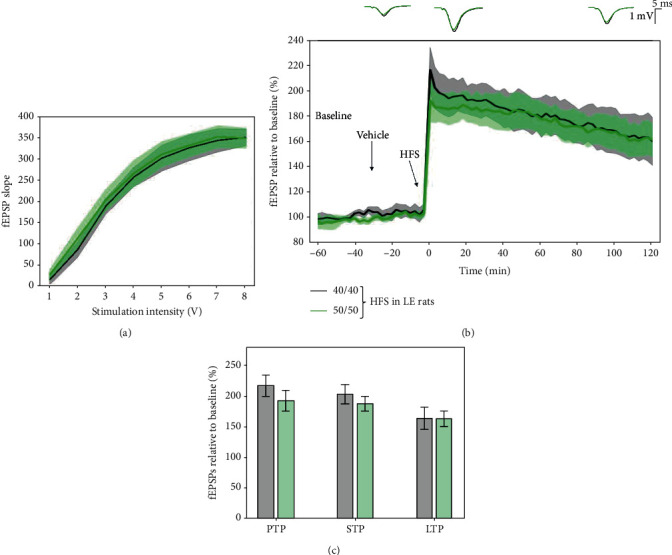
Differential synaptic LTP response induced by the 40/40 and 50/50 HFS protocols in LE rats. (a) Average input/output curves between stimulation voltage and fEPSP slope overlap and show no significant difference in basal synaptic excitability. (b) fEPSPs were enhanced compared to baseline following 40/40 HFS (black, *n* = 8) and 50/50 HFS (green, *n* = 11) to a similar level. LTP induced by HFS was high throughout the whole 2-hour posttetanisation; however, there was no significant difference between the LTP response induced by both 40/40 and 50/50 HFS protocols. Data are presented as means ± 95%confidenceintervals. Outsets above LTP curves represent average waveform field potentials during 30 min baseline prior to tetanisation and 0-30 min and 90-120 min posttetanisation. Horizontal bar: 1 mV; vertical bar: 5 ms. (c) PTP, STP, and LTP responses are presented as means ± 95%confidenceintervals.

No significant differences were found when comparing 40/40 to 50/50 HFS protocols (40/40: 215.663 ± 17.427%; 50/50: 191.45 ± 16.883%; *p* = 0.072), STP (40/40: 202.453 ± 15.719%; 50/50: 187.08 ± 12.348%; *p* = 0.145), and LTP (40/40: 163.374 ± 17.267%; 50/50: 187.08 ± 12.348%; *p* = 0.902) (Figure 3(c)). At the same time, it should be noted that LTP response induced by the 50/50 HFS protocol degraded at a faster rate during the two-hour posttetanisation than the 40/40 one (*p* < 0.001 for differences in slopes in a mixed-effect model).

As there was no substantial effect in lowering the voltage intensity on LTP response in LE rats, neither of the two protocols were used for further pharmacological studies, as they produced a ceiling effect on LTP.

### 3.2. 5-Train TBS Protocol Produced a Submaximal Margin of LTP Response in SD Rats

**Figure 4:**
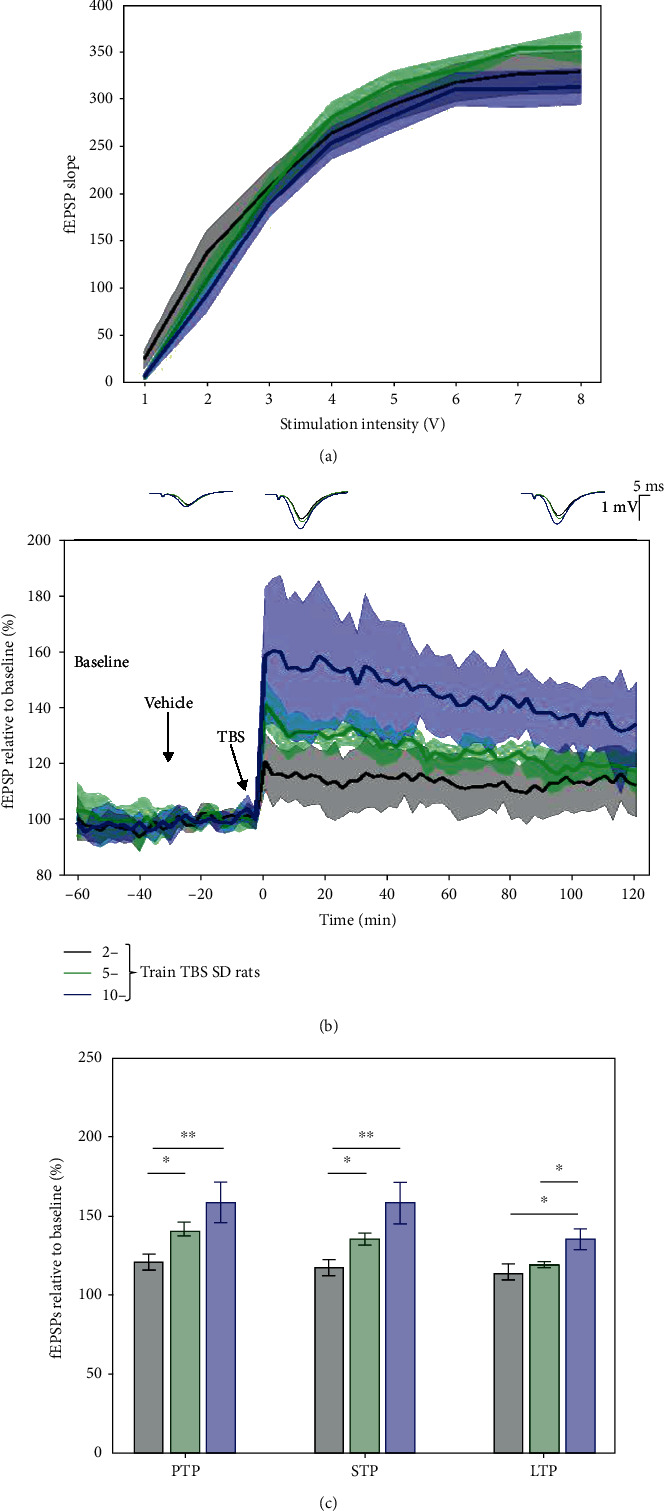
LTP response to different trains of the TBS protocol in Sprague-Dawley rats. As the number of TBS trains were increased, the LTP response increased in the SD rats. (a) Average input/output curves between stimulation voltage and fEPSP slope overlap for the study groups indicating no difference in basal synaptic excitability. (b) fEPSPs were enhanced compared to baseline following 2 trains of TBS (black, *n* = 8), 5 trains of TBS (green, *n* = 7), and 10 trains of TBS (blue, *n* = 6); as the number of trains were increased, the larger was the fEPSP increase. LTP response induced by 10 × TBS was significantly higher than both 2 × TBS and 5 × TBS throughout the whole 2-hour posttetanisation. Data are presented as means ± 95%confidenceintervals. Outsets above LTP curves represent average waveform field potentials during 30 min baseline prior to tetanisation and 0-30 min and 90-120 min posttetanisation. Horizontal bar: 1 mV; vertical bar: 5 ms. (c) PTP, STP, and LTP responses are presented as means ± 95%confidenceintervals. ^∗^*p*value < 0.05 and ^∗∗^*p*value < 0.01.

In a previous study evaluating 2-, 5-, 10-, and 20-train TBS protocols in SD rats, an increased LTP response with the number of trains was found until 20 trains [28]. The 5-train TBS was selected because it offered a window for pharmacological evaluation on LTP response. Similar results were found in the present study comparing 2-, 5-, and 10-train TBS protocols in SD rats. I/O curves of protocols overlapped, confirming that all experiments began with a similar baseline excitability; however, the I/O curves for the 5 × TBS protocol were slightly higher between 6 and 8 V, which did not influence or significantly shift the stimulation intensity and plasticity response (Figure 4(a)).

Our results showed PTP equals to 120.76 ± 9.967% (2 trains), 141.398 ± 8.651% (5 trains), and 158.447 ± 24.71% (10 trains); STP equals to 117.007 ± 9.543% (2 trains), 135.478 ± 7.304% (5 trains), and 158.175 ± 25.525% (10 trains); and LTP equals to 114.036 ± 10.601% (2 trains), 119.011 ± 3.979% (5 trains), and 135.309 ± 12.896% (10 trains). Statistical assessment of differences between different TBS tetanisation trains was for PTP (2 vs. 5 (*p* = 0.012), 2 vs. 10 (*p* = 0.01), and 5 vs. 10 (*p* = 0.231)), STP (2 vs. 5 (*p* = 0.015), 2 vs. 10 (*p* = 0.006), and 5 vs. 10 (*p* = 0.12), and LTP (2 vs. 5 (*p* = 0.46), 2 vs. 10 (*p* = 0.02), and 5 vs. 10 (*p* = 0.03)) (Figure 4(c)). It was also found that with an increase of the number of trains, LTP responses through the 2-hour course after TBS degrade faster (model's slope: 2 vs. 5 (*p* < 0.001), 2 vs. 10 (*p* < 0.001), and 5 vs. 10 (*p* < 0.001), mixed-effect model).

### 3.3. 5-Train TBS Protocol Produced a Submaximal Margin LTP Response in LE Rats

**Figure 5:**
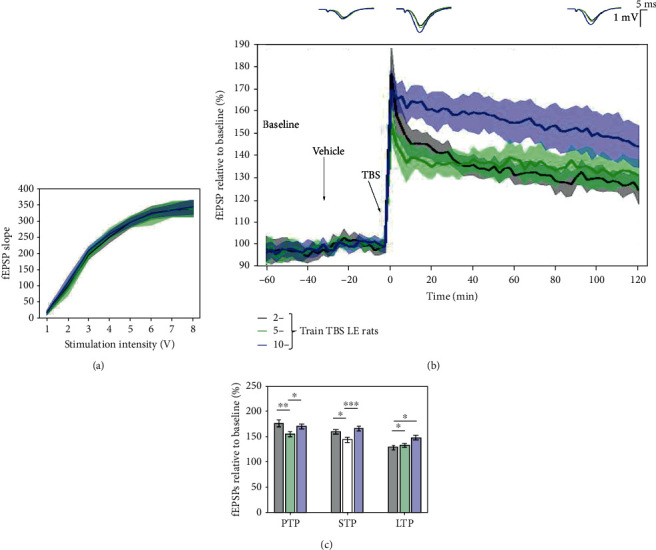
LTP response to different trains of the TBS protocol in LE rats. (a) Collective input/output curves between stimulation intensities and fEPSP slope overlap, showing no significant difference in basal synaptic excitability. (b) When compared to 2 trains of TBS (black, *n* = 11), there was a significant increase in the LTP response induced by 5 trains (green, *n* = 10) and 10 trains (blue, *n* = 9) of TBS. Values represent means ± 95%confidenceintervals. Outsets above LTP curves represent average waveform field potentials during 30 min baseline prior to tetanisation and 0-30 min and 90-120 min posttetanisation. Horizontal bar: 1 mV; vertical bar: 5 ms. (c) PTP, STP, and LTP responses are presented as means ± 95%confidenceintervals. ^∗^*p*value < 0.05; ^∗∗^*p*value < 0.01; and ^∗∗∗^*p*value < 0.001.

This study in LE rats followed the same protocol as the one described for the SD rats above, with an idea of TBS tetanisation protocol validation on another strain of animals and establishing between strain comparison. I/O curves used for all protocols overlapped, confirming no significant difference in basal synaptic excitability (Figure 5(a)). No consistent change in amplitudes of response as a function from the number of trains was found: 177.087 ± 12.516% (2 trains), 154.977 ± 6.952% (5 trains), and 169.873 ± 8.689% (10 trains) for PTP; 159.066 ± 8.053% (2 trains), 144.259 ± 6.802% (5 trains), and 165.996 ± 6.846% (10 trains) for STP; and 128.468 ± 4.014% (2 trains), 132.656 ± 6.241% (5 trains), and 147.82 ± 9.559% (10 trains) for LTP. At the same time, several significant effects of TBS train tetanisation were revealed for PTP (2 vs. 5 (*p* = 0.008), 2 vs. 10 (*p* = 0.38), and 5 vs. 10 (*p* = 0.017)), STP (2 vs. 5 (*p* = 0.016), 2 vs. 10 (*p* = 0.24), and 5 vs. 10 (*p* < 0.0001)), and LTP (2 vs. 5 (*p* = 0.27), 2 vs. 10 (*p* = 0.001), and 5 vs. 10 (*p* = 0.017)). No consistent changes in the rate of LTP response degradation was found, although differences in slopes in linear mixed-effect models were significant (*p* < 0.001 between any pair of trains' numbers).

The 5-train TBS was selected for subsequent pharmacological studies because it offered a window for pharmacological evaluation on LTP response. However, the 2 × TBS protocol could also be selected as both protocols elicited submaximal LTP responses.

**Figure 6:**
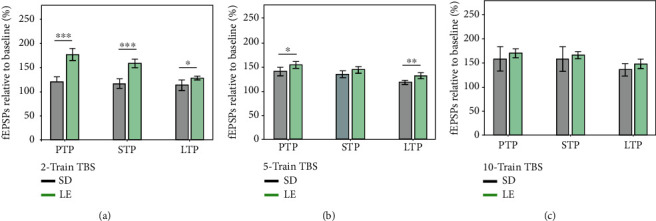
Comparison in PTP, STP, and LTP between SD and LE rats in (a) 2-train TBS, (b) 5-train TBS, and (c) 10-train TBS protocols. Results are shown as means ± 95%confidenceintervals. ^∗^*p*value < 0.05; ^∗∗^*p*value < 0.01; and ^∗∗∗^*p*value < 0.001.

When comparing results of LTP responses to different trains of the TBS protocol between LE and SD rats, the following results were obtained: (2 trains) *p* < 0.001 for PTP, *p* < 0.001 for STP, and *p* < 0.001 for LTP (Figure 6(a)); (5 trains) *p* = 0.032 for PTP, *p* = 0.123 for STP, and *p* = 0.008 for LTP (Figure 6(b)); and (10 trains) *p* = 0.338 for PTP, *p* = 0.501 for STP, and *p* = 0.143 for LTP (Figure 6(c)). When comparing the rate of degradation, it was found that LTP degrades faster in SD rats (*p* < 0.001) for 2-train TBS, and LTP degrades faster in LE rats for 5-train (*p* < 0.001) and 10-train TBS (*p* = 0.009).

### 3.4. Interstimulus Intervals of 30 ms and 50 ms ISIs Produced Similar PPF Response in LE Rats

**Figure 7:**
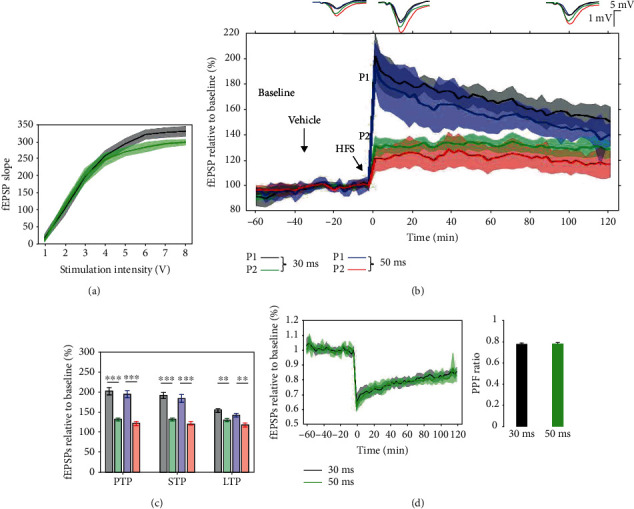
There was no significant difference in the paired-pulse ratio (PPR) using both 50 ms and 30 ms interstimulus intervals (ISIs) for the PPF protocol. (a) Collective input/output curves between stimulation voltage and fEPSP slope show no significant difference in basal synaptic excitability between 0 and 5 V. (b) fEPSP slope relative to baseline for both the first and second responses of both 50 ms (*n* = 11) and 30 ms (*n* = 6) interstimulus intervals show a similar response; the second response (P2) for both is lower than the first response (P1), and there is no significant difference between using 50 ms and 30 ms ISIs. Values represent averages ± 95%confidenceintervals. Outsets above LTP curves represent average waveform field potentials during 30 min baseline prior to tetanisation and 0-30 min and 90-120 min posttetanisation. Horizontal bar: 1 mV; vertical bar: 5 ms. (c) PTP, STP, and LTP responses are presented as means ± 95%confidenceintervals; ^∗∗^*p*value < 0.01 and ^∗∗∗^*p*value < 0.001. (d) The curve shows the normalized ratios of the second and first field potential slopes evoked by paired-pulse stimulation with 30 and 50 ms intervals for all groups. Average PPRs show no significant difference. Right bar plot shows no significant difference in the average PPRs for 2-hour posttetanisation.

The length of interstimulus intervals (ISIs) between paired pulses affects the level of facilitation, and when the length of ISIs is lower than or exceeds a particular level, it will result in a paired-pulse depression. I/O curves used for 30 ms and 50 ms ISI protocols overlapped, confirming no significant difference in basal synaptic excitability (Figure 7(a)). The average paired-pulse ratio (PPR) using 30 ms ISIs during the induction phase (0.661 ± 0.056% of baseline) was like (*p* = 0.453) the PPR using 50 ms ISIs (0.627 ± 0.033% of baseline) (Figure 7(d)). When comparing time PPR between 30 ms and 50 ms ISI protocols throughout the whole 2-hour posttetanisation using a linear mixed-effect model, no significant differences were found in the slope of the model (*p* = 0.293), suggesting similar rate of changes in time. The PPF indexes did not differ either (0.78 ± 0.02 for 30 ms protocol and 0.79 ± 0.02 for 50 ms protocol, *p* = 0.341) (Figure 7(d), right bar plot). The 50 ms ISI protocol elicited a similar margin as previously described [11]; therefore, this protocol was selected for subsequent pharmacological studies.

### 3.5. Input/Output Criteria and Pharmacology in LE Rats

**Figure 8:**
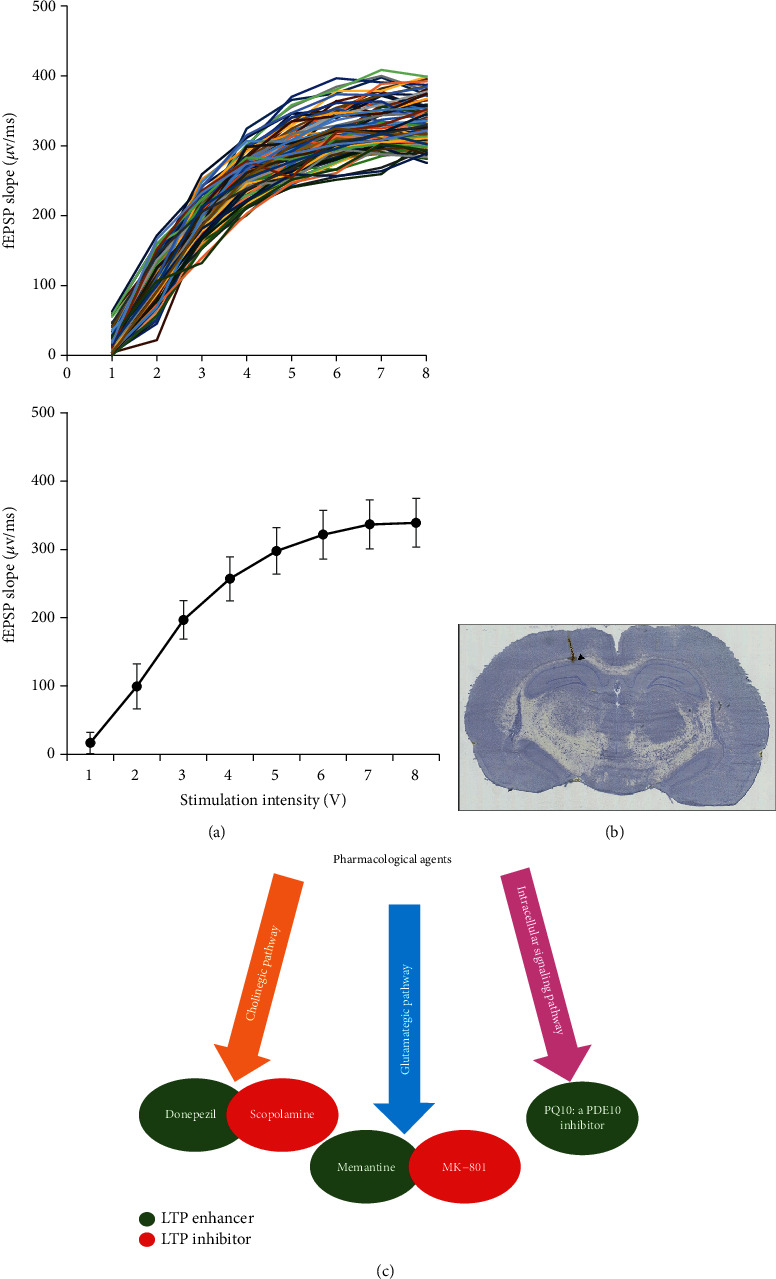
(a) Spaghetti plot of individual input/output curves and average values. Individual fEPSP slopes (*n* = 103) lie between 250 *μ*V/ms and 400 *μ*V/ms at 200 *μ*s stimulus duration, and the calculated test stimulus fits between 3 V and 4 V for all experiments. Value represent mean ± SEM. (b) A representative image depicting stimulation and recording sites. (c) Pharmacological agents used to study LTP response in LE rats.

Prior to an LTP induction protocol, I/O curves were used as the first gate to establish inclusion and exclusion criteria. Stimulation at intensities ranging from 1 to 8 V in steps of 1 V at 0.033 Hz frequency and 200 *μ*s duration were delivered and 3 responses were recorded at each intensity. An individual fEPSP slope lies between 250 *μ*V/ms and 400 *μ*V/ms at 200 *μ*s stimulus duration, and the I/O curves followed a sigmoid curve distribution and the calculated test stimulus fits between 3 V and 4 V for all experiments (Figure 8(a)). In Figure 8(b), a representative image of the site of electrical stimulation and recording verified with standard histology technique is displayed. For the validation of the sensitivity of the TBS protocol in LE rats, three neural transmission mechanisms that are critical in the LTP function were pharmacologically modulated, with the aim of either enhancing or inducing an LTP deficit. Three pathways that were targeted were the cholinergic pathway using donepezil and scopolamine, the glutamatergic pathway using memantine and MK-801, and the intracellular cAMP signaling pathway using the PDE10 inhibitor PQ10 (Figure 8(c)).

### 3.6. Effects of Donepezil on 5 × TBS LTP Response

**Figure 9:**
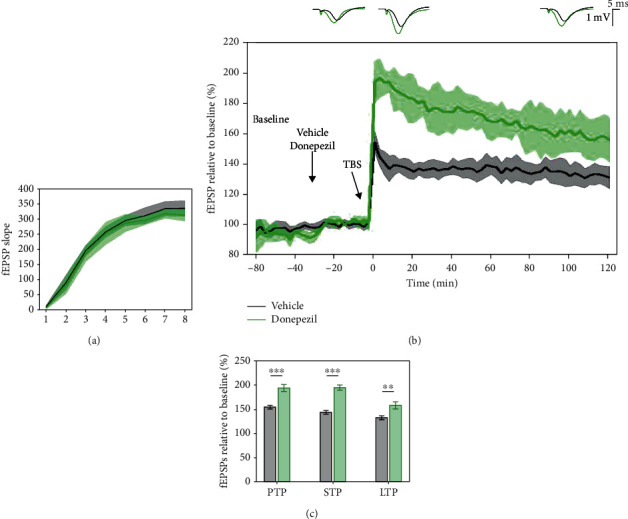
Donepezil (1 mg/kg) significantly enhanced 5 × TBS LTP response when compared to control, saline-treated subjects. (a) Collective input/output curves of stimulation voltage and fEPSP slope overlap and show no significant difference in basal synaptic excitability prior to treatment with donepezil. However, the average response between 9 and 10 V showed a slightly lower response than control subjects in donepezil-treated subjects. (b) Basal fEPSPs were not affected; however, the LTP response of donepezil-treated subjects (green, *n* = 5) was significantly greater than control, saline-treated subjects (black, *n* = 10). Values represent mean ± 95%confidenceintervals. Outsets above LTP curves represent average waveform field potentials during 30 min baseline prior to tetanisation and 0-30 min and 90-120 min posttetanisation. Horizontal bar: 1 mV; vertical bar: 5 ms. (c) PTP, STP, and LTP responses are presented as means ± 95%confidenceintervals; ^∗∗^*p*value < 0.01 and ^∗∗∗^*p*value < 0.001).

To evaluate the sensitivity of the 5 × TBS protocol to increased cholinergic activity in LE rats, donepezil, an acetylcholinesterase inhibitor, was administered 30 minutes before tetanisation. I/O curves showed no major difference in basal synaptic excitability (Figure 9(a)). The significant effect of donepezil was seen on PTP (*p* < 0.001, 154.977 ± 6.953% vehicle vs. 194.294 ± 13.836% donepezil), STP (*p* < 0.001, 144.259 ± 6.802% vehicle vs. 194.866 ± 10.087% donepezil), and LTP (*p* = 0.002, 132.656 ± 6.241% vehicle vs. 158.668 ± 13.078% donepezil) (Figures 9(b) and 9(c)). A linear mixed-effect model revealed a significant effect of donepezil on LTP degradation after the TBS tetanisation (model's slope: *p* < 0.001). The changes in the LTP response throughout the 2-hour posttetanisation demonstrate a sensitivity of the 5 × TBS protocol to increases in cholinergic activity in LE rats.

### 3.7. Effects of Scopolamine on 5 × TBS LTP Response

**Figure 10:**
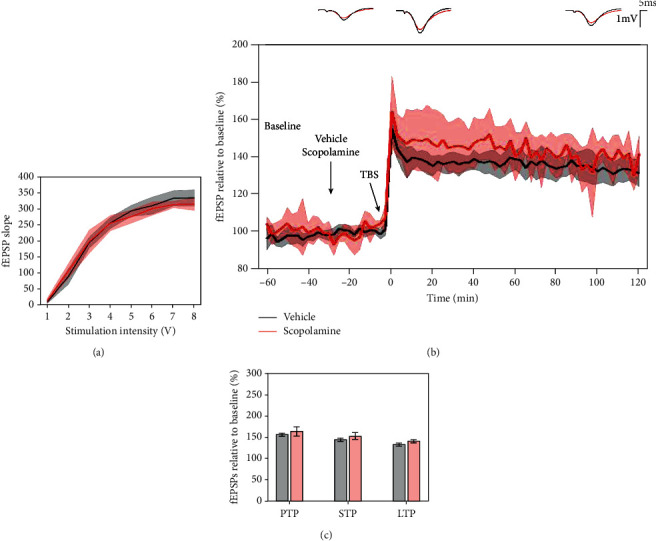
Scopolamine (0.64 mg/kg) had no significant effect on the LTP response in LE rats. (a) Collective input/output curves between stimulation voltage and fEPSP slope overlap and show no significant difference in basal synaptic excitability. (b) Both scopolamine (red, *n* = 7) and control, vehicle-treated (black, *n* = 10) subjects had an increase in fEPSP in response to 5 × TBS tetanisation; however, there was no significant difference in the LTP response throughout the 2-hour posttetanisation. Values represent means ± 95%confidenceintervals. Outsets above LTP curves represent average waveform field potentials during 30 min baseline prior to tetanisation and 0-30 min and 90-120 min posttetanisation. Horizontal bar: 1 mV; vertical bar: 5 ms. (c) PTP, STP, and LTP responses are presented as means ± 95%confidenceintervals.

To test the sensitivity of the 5 × TBS protocol to reduced cholinergic activity in LE rats, scopolamine, an antimuscarinic was administered 30 minutes before tetanisation. I/O curves showed no major difference in basal synaptic excitability (Figure 10(a)). No significant effect of scopolamine was seen either on PTP (*p* = 0.304, 154.977 ± 6.952% vehicle vs. 164.183 ± 18.831% scopolamine), or on STP (*p* = 0.227, 144.259 ± 6.802% vehicle vs. 152.904 ± 13.258% scopolamine), or on LTP (*p* = 0.172, 132.656 ± 6.241% vehicle vs. 139.704 ± 6.647% scopolamine) (Figure 10(c)). A mixed-effect model did not reveal a significant effect of scopolamine on LTP degradation either (model's slope: *p* = 0.3).

### 3.8. Effects of Memantine on 5 × TBS LTP Response

**Figure 11:**
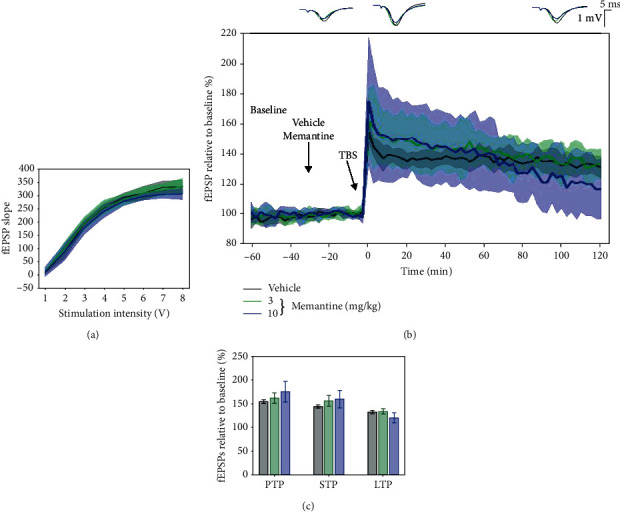
Memantine (3 and 10 mg/kg) had no significant effect on the 5 × TBS-induced LTP response in LE rats. (a) Collective input/output curves between stimulation voltage and fEPSP slope overlap and show no significant difference in basal synaptic excitability. (b) A slight enhancement of LTP response was observed following both doses of memantine; however, the changes did not reach a significant level (green and blue, *n* = 6 and *n* = 7, respectively) as compared to vehicle-treated animals (black, *n* = 10). Values represent means ± 95%confidenceintervals. Outsets above LTP curves represent average waveform field potentials during 30 min baseline prior to tetanisation and 0-30 min and 90-120 min posttetanisation. Horizontal bar: 1 mV; vertical bar: 5 ms. (c) PTP, STP, and LTP responses are presented as means ± 95%confidenceinterval.

The effect of memantine at 3 and 10 mg/kg on hippocampal fEPSPs was evaluated after 5 × TBS tetanisation. I/O curves were not significantly different, confirming an overall similar basal excitability of the SC-CA1 synapses (Figure 11(a)). No significant difference was found between vehicle, 3 mg/kg, and 10 mg/kg on PTP (154.977 ± 6.952%, 162.008 ± 21.84%, and 175.481%, respectively), STP (144.259 ± 6.802%, 156.165 ± 21.538%, and 160.17 ± 35.734%, respectively), and LTP (132.656 ± 6.241%, 134.111 ± 11.156%, and 120.405 ± 20.626%, respectively) (Figure 11(c)). At the same time, degradation of fEPSPs was different (vehicle vs. 3 mg/kg: *p* < 0.001; vehicle vs. 10 mg/kg: *p* < 0.001; and 3 mg/kg vs. 10 mg/kg: *p* < 0.001, using a linear mixed-effect model).

### 3.9. Effects of MK-801 on 5 × TBS LTP Response

**Figure 12:**
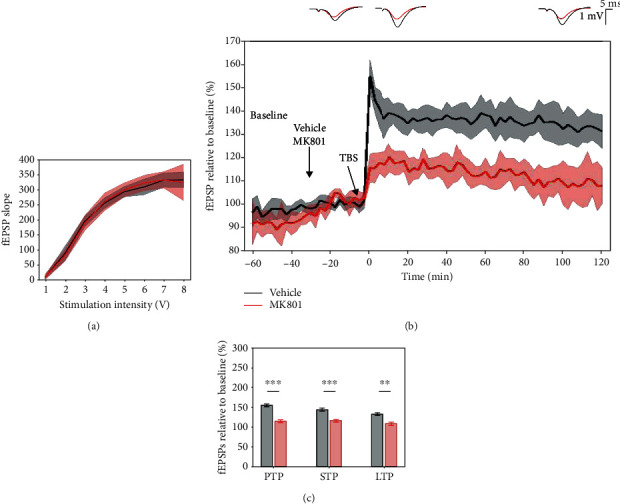
Acute treatment with MK-801 (5 mg/kg) significantly impaired the 5 × TBS LTP response compared to controls. (a) Collective input/output curves between stimulation voltage and fEPSP slope overlap, showing no significant difference in basal synaptic excitability. (b) When compared to vehicle-treated subjects (black, *n* = 10), acute treatment with MK-801 (red, *n* = 7) significantly reduced the 5 × TBS LTP response throughout the whole 2-hour posttetanisation. Values represent means ± 95%confidenceintervals. Outsets above LTP curves represent average waveform field potentials during 30 min baseline prior to tetanisation and 0-30 min and 90-120 min posttetanisation. Horizontal bar: 1 mV; vertical bar: 5 ms. (c) PTP, STP, and LTP responses are presented as means ± 95%confidenceinterval; ^∗∗^*p*value < 0.01 and ^∗∗∗^*p*value < 0.001.

The sensitivity of the 5 × TBS protocol to decreases in glutamatergic transmission in LE rats has been evaluated on the LTP response after acute treatment with MK-801 (5 mg/kg), and I/O curves showed no major difference in basal synaptic excitability (Figure 12(a)). A significant effect of MK-801 was seen on PTP (*p* < 0.001, 154.977 ± 6.952% vehicle vs. 115.284 ± 5.994% MK-801), STP (*p* < 0.001, 144.259 ± 6.802% vehicle vs. 116.231 ± 5.304% MK-801), and LTP (*p* = 0.001, 132.656 ± 6.241% vehicle vs. 108.813 ± 8.577% MK-801) (Figure 12(c)). At the same time, no significant effect of MK-801 was found in the speed of LTP degradation (*p* = 0.335). The results suggest a sensitivity of the 5 × TBS protocol to reduced glutamatergic activity in LE rats.

### 3.10. PQ10 Enhanced LTP Response Elicited with 5 × TBS Protocol

**Figure 13:**
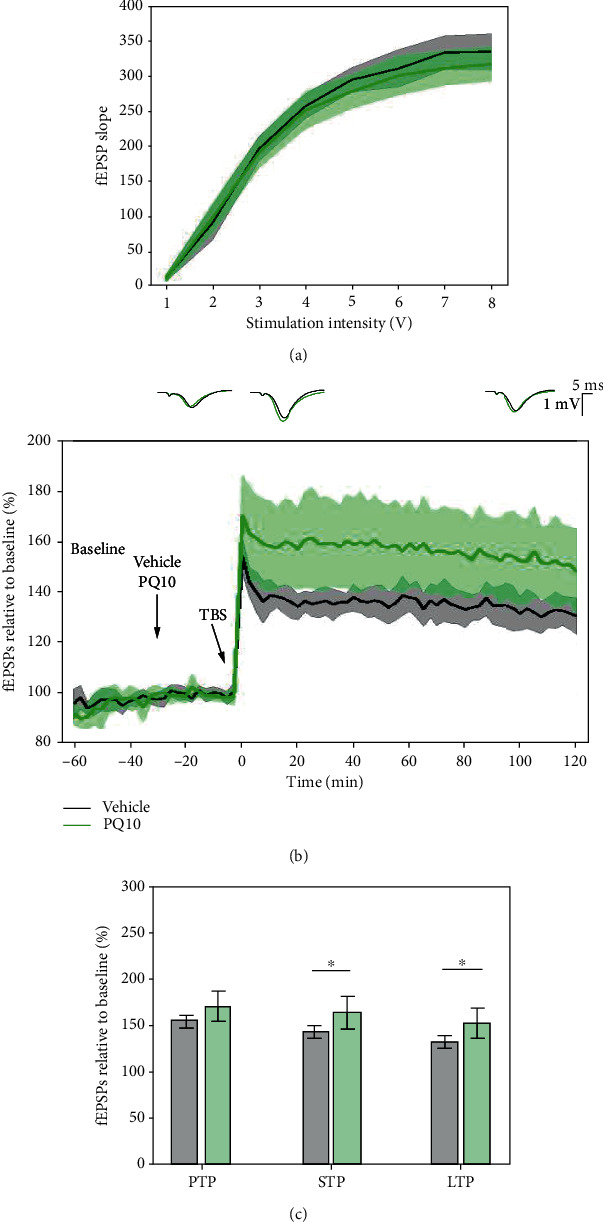
PQ10 enhanced the LTP response to the 5 × TBS protocol. (a) Input/output curves showing no significant differences among groups. (b) PQ10-treated (green, *n* = 8) subjects had increased fEPSPs during the 2-hour posttetanisation recording as compared to control (black, *n* = 10). Values represent means ± 95%confidenceintervals. Outsets above LTP curves represent average waveform field potentials during 30 min baseline prior to tetanisation and 0-30 min and 90-120 min posttetanisation. Horizontal bar: 1 mV; vertical bar: 5 ms. (c) PTP, STP, and LTP responses are presented as means ± 95%confidenceinterval; ^∗^*p*value < 0.05.

To evaluate the sensitivity of the 5 × TBS protocol to increases in cAMP and cGMP activity in LE rats, PQ10 (3 mg/kg), a phosphodiesterase-10 inhibitor, was administered 30 minutes before tetanisation. There was no difference in basal fEPSP levels and therefore no effect on basal synaptic transmission (Figure 13(a)). A significant effect of PQ10 was found on STP (*p* = 0.045, 144.259 ± 6.802% vehicle vs. 160.4 ± 15.217% PQ10) and LTP (*p* = 0.043, 132.656 ± 6.241% vehicle vs. 148.863 ± 14.818% PQ10), but not on PTP (*p* = 0.134, 154.977 ± 6.952% vehicle vs. 166.564 ± 14.228% PQ10) (Figure 13(c)). A mixed-effect model did not reveal a significant effect of PQ10 on LTP degradation (model's slope: *p* = 0.144).

### 3.11. PQ10 Had No Effect on the Presynaptic Form of LTP

**Figure 14:**
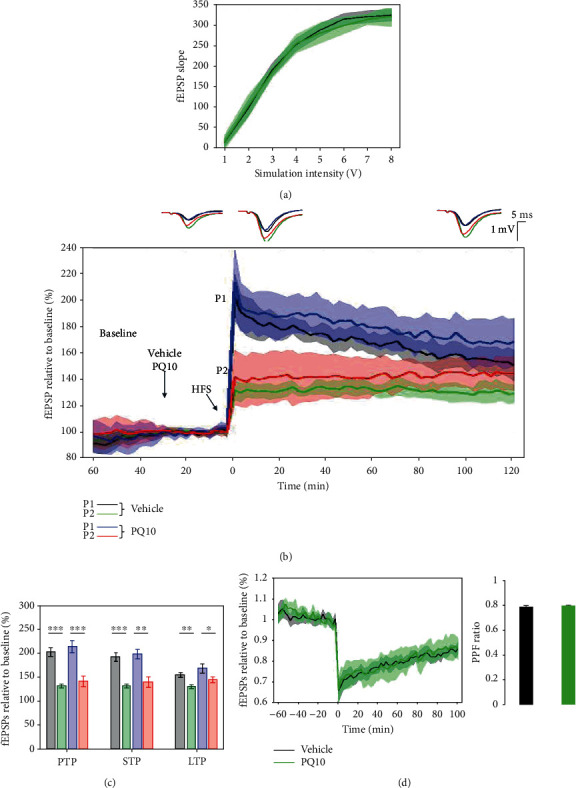
PQ10 did not affect field postsynaptic potentials (fEPSP1 and fEPSP2) and paired-pulse facilitation (PPF) recorded at the SC-CA1 synapse during LTP. (a) Collective input/output curves between stimulation voltage and fEPSP slope overlap, showing no significant difference in basal synaptic excitability. (b) PQ10 (*n* = 6) did not alter the induction and maintenance of LTP response measured from the slope of the fEPSP1 (blue) and fEPSP2 (red) as compared to vehicle-treated subjects (black and green, respectively, *n* = 10). Values represent averages ± 95%confidenceintervals. Outsets above LTP curves represent average waveform field potentials during 30 min baseline prior to tetanisation and 0-30 min and 90-120 min posttetanisation. Horizontal bar: 1 mV; vertical bar: 5 ms. (c) PTP, STP, and LTP responses are presented as means ± 95%confidenceintervals. ^∗^*p*value < 0.05; ^∗∗^*p*value < 0.01; and ^∗∗∗^*p*value < 0.001. (d) No difference in the paired-pulse facilitation of PQ10-treated and vehicle-treated subjects. The bar plot showing average PPR values during the 2-hour posttetanisation showed no significant difference between PQ10- and vehicle-treated subjects.

The effects of PQ10 (3 mg/kg) on the PPF response in LE rats showed no consistent change in the presynaptic form of LTP, suggesting an effect on the loci of LTP at the postsynaptic level. Collective I/O curves for both groups overlap, showing no significant difference in basal synaptic excitability in the recorded SC-CA1 synapses (Figure 14(a)).

The average paired-pulse ratio (PPR) of PQ10-treated subjects during the induction phase (0.663 ± 0.06% of baseline) was not significantly different from the control subjects (0.661 ± 0.056% of baseline) (Figure 14(d)). When comparing time PPR between subjects treated with PQ10 and vehicle throughout the whole 2-hour posttetanisation using a linear mixed-effect model, no significant differences were found in the slope of the model (*p* = 0.393), suggesting a similar rate of changes in time.

## 4. Discussion

The present study evaluated the margin of plasticity response to three LTP protocols (HFS, TBS, and PPF) at the SC-CA1 hippocampal circuit in anesthetized LE rats, which is a commonly used strain in behavioral cognitive tests. The 50/50 HFS protocol, widely used in other strains such as SD rats, induced a ceiling effect of the LTP response in LE rats, highlighting strain differences in synaptic plasticity. The 2-train TBS protocol significantly enhanced the LTP response in LE rats compared to the 5- and 10-train TBS protocols. The PPF protocol, which gives an indication of the loci of LTP (pre- or postsynaptically), showed no significant difference in plasticity responses with varying lengths of interstimulus intervals between paired pulses.

### 4.1. LTP Response Was Significantly Greater in LE Rats than in SD Rats

Long-lasting plasticity is expressed sequentially in phases over time. Posttetanic potentiation is mostly considered to be of presynaptic origin and lasts between 30 s and a few minutes [29]. Application of brief stimuli to the Schaffer collateral fiber elicits three different phases such as PTP, STP, and LTP. Presynaptic accumulation of Ca^2+^ causes PTP that readily decreases after Ca^2+^ clearance. In this phase, PTP is NMDA receptor independent. By contrast, the following STP and LTP phases are of postsynaptic origin and are NMDA receptor-dependent forms of potentiation [30–33].

HFS induction LTP protocol falls under the non-theta-like pattern of neuronal excitability, which consists of 200 Hz stimulation that is often repeated with intervals up to several seconds long [34]. The 200 Hz HFS protocol used in the present work is based on a previously developed protocol [27], which is widely used in both Wistar and SD rat strains to evaluate the potency of drugs to influence synaptic plasticity. The present study used LE rats, which is the mostly used strain in cognitive behavioral tasks as they are known to have advanced synaptic plasticity efficiency compared to other strains. As expected, the average 50/50 HFS LTP response of the SC-CA1 synapses in anesthetized LE rats were significantly higher, up to the ceiling effect on LTP levels compared to that in anesthetized SD rats. Intriguingly, HFS did not induce posttetanic potentiation in LE rats as it was observed in SD rats. Thus, although this protocol is widely used in the SD rat strain, weaker stimulation protocols are required for pharmacological studies in LE rats. Previous research has shown that anesthetized LE rats display a higher LTP response in the medial perforant pathway of the DG than anesthetized SD rats [23]. The significant difference in the HFS plasticity response between LE and SD provides further support that LE rats have more excitable SC-CA1 synapse and enhanced synaptic plasticity than SD rats, which research has shown to be due to differences in learning ability, expression of receptors, and/or expression of immediate early genes [3].

Questions have been raised about whether a similar response in the magnitude of LTP might occur using a 50/50 HFS protocol or a slightly weaker 40/40 HFS protocol, whereby the voltage used throughout the experiment was at 50% of the maximal voltage stimulation intensity obtained in the I/O curve. The data we have presented show that the stimulation protocol with the 40/40, which only accounted for 10% difference from the 50% stimulation intensity, produced similar potentiation. It may be the case that the lower limit of 30% of the voltage stimulation in the linear range of the I/O curve might influence how synapses respond to the priming episode of tetanisation and reveal significant differences in the magnitude of LTP, but this question requires further study.

As both HFS protocols did not elicit a suitable margin to evaluate the effects of drugs on synaptic facilitation for LTP in LE rats, the TBS protocol was evaluated.

TBS induction LTP protocol has gained interest in recent years because it is believed to approximate a physiologically relevant pattern of excitability and to mimic endogenous theta-frequency EEG activity (4–8 Hz) recorded in the hippocampus when the animal is engaged in learning and memory functions. TBS is a repeating pattern of short bursts of pulses (e.g., 4 pulses at 100 Hz) with brief pauses (∼200 ms) between bursts [34–37]. Studies have shown that the two protocols engage different biochemical pathways to produce differences in LTP magnitude and time course kinetics [38–40]. Because the magnitude of LTP is affected by frequency, number of pulses, and stimulus intensity during tetanus, it was expected that incremental changes in the number of TBS trains would produce proportional increases in the magnitude of synaptic potentiation since LTP response increases with stronger stimulation parameters, e.g., LTP response may be greater for the 10 × TBS than for the 5 × TBS and 2 × TBS treatments as previously described [28].

In SD rats, PTP responses, characterized by a large increase in fEPSP slope post-TBS followed by a sharp decline in fEPSP slope size, showed increases with TBS trains. There was no significant difference between the average LTP response induced by 2 × TBS and 5 × TBS; however, the 10 × TBS protocol induced a significantly higher average LTP response. In LE rats, the 5 and 10 × TBS produced weaker PTP response when compared with the 2 × TBS protocol. These differences seem to suggest that PTP requires a rigorous stimulation protocol in order to be induced in this projection and that 10 × TBS was slightly weak in producing greater PTP than the other protocols. The 10 × TBS protocol produced an initial slow rise and gradual peaking in the early and late phases of the LTP response, which was different compared to the 2 × TBS and 5 × TBS protocols. The results suggest that the magnitude of PTP and LTP is not sensitive to a linear increase in train pulse number.

Since there was a slight difference between the results obtained with the 5 × TBS submaximal protocol in LE compared to SD rats here or published earlier [28], this protocol has been selected for pharmacological studies in LE rats.

To determine the loci of LTP (pre- or postsynaptically) in anesthetized LE rats, a paired-pulse facilitation protocol (PPF) was evaluated. PPF is a paradigm for synaptic short-term plasticity with a mostly presynaptic origin [17, 32, 33], consisting of paired pulses delivered before and after the 200 Hz HFS protocol. When the synapse is activated twice in rapid succession, the magnitude of the second response should be larger due to greater facilitation of neurotransmitter release, caused by residual calcium when the basal release probability (*p*) is low. In contrast, if *p* is high in basal conditions, the consequence is a paired-pulse depression [41, 42]. The paired-pulse ratio (PPR) is the second fEPSP response divided by the first fEPSP response and determines the probability of vesicular release. The lower the PPR, the greater the probability of neurotransmitter release [41]. Previous research has highlighted the significance of changing the interstimulus intervals (ISIs) between paired pulses on the level of facilitation. The level of LTP facilitation at increasing intervals (20-100 ms) in the CA1 region of Wistar rat hippocampal slices revealed that the maximal levels of facilitation were between 50 and 100 ms [17]. Short ISIs induced paired pulse depression, whilst ISIs above 800 ms were ineffective at evoking synaptic plasticity. An ISI value of 50 ms was selected for this study as it was expected to give a suitable dynamic range [17]. This study compared the use of 30 ms and 50 ms ISIs in the CA1 region of anesthetized LE rats and found that there was no significant difference in the PPFs and the levels of facilitation when using both ISIs. It was therefore concluded that the range of ISIs was wider in anesthetized LE rats in comparison to the previous study; therefore, 50 ms ISIs were selected for further pharmacological studies, as this coincided with previous studies.

The cholinergic and glutamatergic systems have been reported to play a crucial role in cognitive functions [43, 44]. The levels of hippocampal acetylcholine and glutamate decline with the aging process, and the disturbance of cholinergic and glutamatergic transmission is associated with cognitive impairments and the onset of neurodegenerative disease such as AD [43–46]. Synaptic dysfunction in AD has been presumed to be related to damaged cholinergic and glutamatergic receptors following accumulation of extracellular *β*-amyloid plaques and pathological neurofibrillary tangles [45–48].

Acetylcholinesterase inhibitors such as donepezil attenuate cognitive deficits in mild and moderate AD patients [49–51]. NMDARs have also been reported to be involved in Alzheimer's disease [48]. Memantine is a moderate-affinity uncompetitive antagonist of glutamate NMDA receptors. It is licensed for use in mild, moderate, and severe AD [52]. It has been reported that memantine could provide both neuroprotection and symptomatic improvement through rapid, moderate-affinity, voltage-dependent NMDAR channel blockade [53, 54].

PDEs degrade the intracellular second messengers cAMP and/or cGMP and terminate intracellular signaling that leads to CREB activation. Accumulating preclinical evidence indicates that PDE inhibitors promote learning and memory, and inhibition of PDE10A has been suggested as a promising therapeutic strategy for psychiatric and neurodegenerative diseases, based on its efficacy in animal models of schizophrenia and AD [55]. We hypothesized that a PDE10A inhibitor would promote plasticity response in LE rats.

To gain more insight into the sensitivity of the selected protocols, the mechanistic roles of three critical pathways for LTP induction and persistence were pharmacologically targeted (Figure 8(c)): (a) the cholinergic transmission through muscarinic receptor blockade and extracellularly acting AChE, (b) the glutamatergic transmission using an NMDA partial agonist and antagonist, and (c) the intracellular signaling using a phosphodiesterase inhibitor. All of these pathways interfere with learning and memory.

### 4.2. Donepezil, an Acetylcholinesterase Inhibitor, Significantly Enhanced the LTP Response, Whereas Scopolamine, a Nonspecific Muscarinic Antagonist, Had a Subtle Effect, Demonstrating the Sensitivity of the TBS Protocol to Enhanced Cholinergic Tone

Modulation of cholinergic receptors may affect LTP response through disinhibition of CA1 pyramidal cells [56] or enhancement of NMDA currents [57]. The facilitation of hippocampal LTP has been shown by pharmacological and nonpharmacological experimental manipulations of acetylcholine levels. In vitro application of cholinergic agonists enhanced hippocampal LTP [58–60], and stimulation of the medial septal cholinergic pathway enhanced hippocampal LTP response in vivo [61]. The acetylcholinesterase inhibitor donepezil, which is used in the treatment of mild-to-moderate AD [62] by preventing the breakdown of acetylcholine, was reported to extend the duration of LTP in the DG of old animals [63]. The sensitivity of the 5 × TBS protocol was evaluated based on previous research in rat hippocampal slices, which showed that donepezil was able to significantly enhance the LTP response in the SC-CA1 pathway within a specific concentration range of 0.1-0.5 *μ*M [64], particularly in the early phase of LTP, delaying the decay of LTP. In the present study, 1 mg/kg of donepezil was able to significantly enhance the LTP response in anesthetized LE rats, when compared to control, saline-treated subjects. The significance of LTP increase compared to controls declines rapidly over the 2-hour posttetanisation, which confirms earlier findings which suggests that donepezil's effects on cholinergic modulation are more potent in the early phase of LTP [62]. Previous research proposes that donepezil facilitates LTP mechanisms by enhancing the NMDA-induced current, facilitating LTP via enhanced muscarinic and nicotinic receptor activation and its interaction with *σ*1 receptors, a protein in the endoplasmic reticulum, which modulates calcium signaling through the IP3 receptor [64]. We report here that donepezil was capable of enhancing and hence prolonging early LTP into late LTP in the SC-CA1 pathway in anesthetized animals. This supports previous findings showing that donepezil is able to delay the decay of LTP in vivo [63].

In previous studies, muscarinic blockade or lesion of the hippocampal cholinergic neurons did not affect hippocampal LTP [65–67]. However, scopolamine can suppress the expression of LTP in both ex vivo [68] and in vivo [63] models. In the latter in vivo study, a higher dose of scopolamine (5 mg/kg) was reported to impair the behavioral enhancement of LTP but did not abolish LTP per se, supporting the hypothesis that cholinergic inputs impinging on muscarinic receptors in the hippocampus are responsible for the larger LTP induced during walking than during immobility [69]. In a seminal study, scopolamine was able to impair LTP response in anesthetized rats, demonstrating the critical involvement of the cholinergic drive to the hippocampus, in the control of LTP induction, relying on the activation of muscarinic receptors [70]. This later study used a higher dosage of scopolamine (0.64 mg/kg) to reveal a marginally insignificant scopolamine treatment effect throughout the 2-hour posttetanisation when compared to saline-treated subjects. The study by Ovsepian [70] targeted the basal region of the CA1 “Stratum Oriens,” whilst this study targeted the apical region, the stratum radiatum. A few reports suggest that excitatory inputs to the basal rather than apical synapses of the CA1 pyramidal cells are more favourably inclined to cholinergic enhancement of LTP [71–75]. Accordingly, the effects of scopolamine on CA1 basal and apical LTP responses were compared, and found that LTP was significantly reduced in the basal but not the apical synapses when compared to saline control subjects. The limited effect of scopolamine on apical synapses has been proposed to result from multiple factors, including the concentration of cholinergic innervations of septal origin being more to the CA1 basal region rather than the apical region, tetanisation of the basal pathway leading to increased release of acetylcholine with glutamate which induces a stronger depolarization, and finally acetylcholine potentiating the NMDA current through muscarinic receptor activation with basal synapses relying more on NMDA receptor activation and therefore enhancing scopolamine's inhibitory effect [71]. It is hypothesized that the low dose of scopolamine used in the present study did not completely block the cholinergic inputs affecting muscarinic receptors in the hippocampus to alter LTP response. In addition, the limited cholinergic input to the apical region of the CA1 to induce LTP deficit, calls for experiments targeting the basal region in future studies.

Nicotinic modulation influences the balance between excitation and inhibition, and therefore, it participates in the homeostatic regulation of synaptic strength affecting the induction of LTP. Nicotine has been shown to facilitate the induction of LTP at the SC-CA1 pathway through attenuating inhibitory influences on NMDA responses [72] and reversing the cholinergic lesion-induced deficit of the LTP induction in rats [73]. In addition, contextual fear learning enhances the strength of inhibitory synapses on hippocampal pyramidal CA1 neurons, through the activation of nicotinic cholinergic receptors [74], likely of the alpha2-containing nAChR subtype [75]. Future experiments will evaluate effects of the negative modulation of nicotinic receptors on LTP response in LE rats.

### 4.3. Memantine, the Low- to Moderate-Affinity, Uncompetitive Antagonist at the NMDA Receptor, Had No Effect on LTP Response Likely Associated with a Weaker TBS Protocol; However, MK-801, a Noncompetitive NMDA Receptor Antagonist, Significantly Reduced LTP Response

In order to test the sensitivity of the 5 × TBS LTP response to modulation of glutamatergic activity, memantine, the uncompetitive NMDA antagonist was administered 30 minutes prior to TBS. Memantine at a dose of 3 mg/kg had no significant effect on the 5 × TBS LTP response, which may have been a result of the use of a weaker TBS protocol. In comparison to previous research, a therapeutic dose of memantine (<10 mM) has been shown to decrease or not change hippocampal LTP in brain slices perfused with a normal medium in vitro [76, 77]. In support to the present findings, intraperitoneal administration of memantine (10 mg/kg) did not affect the 200 Hz train-induced LTP in CA1 of urethane-anesthetized rats [78]. It is interesting that the blockade of NMDA receptors did not affect SC-CA1 LTP, which has been demonstrated to be NMDA-dependent. Memantine is an open-channel trapping blocker, which enters the channel and blocks current flow only after channel opening. Besides blocking NMDA receptors, memantine affects many neural targets including serotonin, dopaminergic, nicotinic, sigma-1 receptor, and voltage-activated channels [79]. Additional mechanisms of action that may account for the lack of effect on LTP response could be related to the transitions among blocked states of a receptor. Accordingly, channel-trapping blockers can partially inhibit, have no effect, or encourage channel closure.

In rats, the administration of the noncompetitive NMDA antagonist, MK-801, has been shown to induce short-lasting, psychotic behavior followed by long-term impairments in both spatial memory and LTP [10]. As the main receptor involved in LTP induction at the SC-CA1 pathway is the NMDA receptor, blockade of the receptor by MK-801 induced a significant deficit of LTP response, demonstrating the sensitivity of the TBS protocol to NMDA receptor dysfunction. Similar deficits in the LTP response have been observed at the SC-CA1 [80] and perforant path-dentate gyrus synapses of the hippocampus in freely moving rats after acute treatment with MK-801 [79, 81, 82], and impairments have been followed for weeks after a single injection of MK-801 in Wistar rats [10, 82]. The difference in results between some of those studies may result from the targeted path, e.g., DG, whilst this study targeted the SC-CA1 pathway, which may respond differently to NMDA receptor inhibition. There were different stimulation protocols and targeted paths of the hippocampal connectivity used: the previous study used an HFS protocol whilst the present study used TBS. Finally, the difference in rat strains may have influenced individual response to MK-801; the previous study used Wistar rats which are known to have less efficient synaptic plasticity mechanisms in comparison to LE rats used here. Based on the results of the acute MK-801 study, the 5 × TBS protocol showed sensitivity to reduced glutamatergic activity.

### 4.4. PQ10 Significantly Enhanced the 5 × TBS LTP Response, but Did Not Change the PPF Response, Suggesting More Post- than Presynaptic Effect

PDE inhibitors have been proposed as an alternative approach for the treatment of cognitive deficits, and they showed efficacy to enhance memory performance in animal [82] cognition models [82–88]. PDE10A is expressed in multiple brain regions of mammalian species, including the striatum, hippocampus, and cortex [89]. PDE10A inhibitors have been shown to upregulate cAMP and cGMP concentrations in the striatum [90, 91] and hippocampus [92]. The sensitivity of the 5 × TBS protocol was evaluated using PQ10, a PDE10 enzyme with dual specificity for preventing the breakdown of both cyclic adenosine monophosphate (cAMP) and cyclic guanosine monophosphate (cGMP), critical in the latter, protein synthesis-dependent stage of LTP [93]. Whilst most research selectively targeted cAMP through the PDE4 inhibitor rolipram, PDE10 has only very recently become a target for enhancing cognition [93]. Previous studies have demonstrated that rolipram facilitates LTP in the CA1 region of rat hippocampal slices and in anesthetized rats [94], as well as to rescue LTP deficit 1 week after acute administration of MK-801 [10]. Less studies have examined the effect of PDE10 inhibition on LTP response whilst it has become increasingly important to understand the effects of both cAMP and cGMP on synaptic plasticity, as phosphodiesterase enzyme activity targeting both signaling molecules is known to be enhanced in both aging and neurodegenerative diseases such as AD [9]. In this study, the effect on PTP did not reach a significant level.

Inhibition of PDE10 had little effect on PTP, whereas PQ10 significantly enhanced the 5 × TBS STP and LTP response. This process requires more time than the immediate PTP changes, as cAMP and cGMP intracellular signaling pathways induce changes to the synapse leading to the activation of CREB which facilitates the transcription of CRE-dependent genes, requiring protein synthesis [9].

The PPF paradigm has been used to investigate the effects of PDE2A and PDE9A on presynaptic STP in the CA1 region of acute hippocampal slices [11]. The inhibition of PDE2A enhanced STP, whilst the inhibition of PDE9A had no effect. PDE2A is expressed more presynaptically, functions as an integrating element for the crosstalk between the cAMP and cGMP signaling pathways, and plays a key role in determining STP. PDE9A specifically targets cGMP and is concentrated more within the cell bodies and dendrites [11]. As PDE10 plays a similar role to PDE2A in targeting both signaling pathways, it is important to determine where the PDE10 enzyme activity is more concentrated and whether inhibition of PDE10 could modulate presynaptic STP. PQ10 had no significant effect on the PPR as compared to control subjects. As PQ10 enhanced the 5 × TBS LTP response, it failed to change the PPF response, suggesting that PQ10 has more postsynaptic, long-term effects on LTP.

## 5. Conclusion

Different laboratories often use slightly different conditions for LTP experiments, which implies a somewhat limited comparability and replicability of results. In LTP experiments, the details of measurement conditions are important [95], and therefore, the comparison of LTP results from different laboratories is valid at the qualitative mechanistic level. Under nonpharmacological conditions, we have evaluated three LTP induction protocols in anesthetized LE rats: HFS, TBS, and PPF. The results obtained in this study are comparable, and alignment with other studies is only warranted after a careful comparison of the experimental conditions. Both the 50/50 and 40/40 HFS protocols induced a ceiling effect on LTP and were significantly greater than the average response in SD rats, indicating that a weaker protocol would be acceptable for pharmacological evaluation in the LE rat strain. The 5 × TBS protocol elicited a submaximal LTP response, and therefore a suitable margin for pharmacological testing in both SD and LE rats. Finally, the 50 ms ISI PPF protocol was selected to study the locus of LTP response in anesthetised LE rats. Under pharmacological conditions, important pathways in the LTP mechanisms were targeted to assess the sensitivity of the selected paradigms to drugs. First, the cholinergic pathway was targeted using the muscarinic antagonist scopolamine; however, no significant effect on LTP response was found with the dose tested likely due to the increased cholinergic input and activity to the basal region of the CA1 rather than the apical CA1 region, which was targeted in this study. Donepezil significantly enhanced the LTP response, demonstrating the sensitivity of the 5 × TBS protocol to enhanced cholinergic tone. Second, the glutamatergic pathway targeted acutely with MK-801 significantly impaired the LTP response in anesthetised LE rats, demonstrating the sensitivity of the paradigm to changes in NMDA receptor activity. Interestingly, memantine did not have any effect in SC-CA1 LTP, even though it has been demonstrated to be NMDA dependent. Finally, inhibition of cAMP and cGMP intracellular signaling pathways with the PDE10 “PQ10” consistently enhanced the 5 × TBS LTP response but did not change the PPF response, suggesting a locus of LTP at the postsynaptic level. Overall, the present work highlights strain difference, replicates earlier synaptic plasticity results, and reports effective protocols that generate a relatively subthreshold margin of LTP induction and maintenance, which are suitable for pharmacology in the LE rat strain primarily used in cognitive studies.

## Data Availability

All relevant data within the paper are fully available.
